# GABA_B_ receptors negatively modulate excitatory plasticity at the mossy fiber synapse onto parvalbumin-expressing basket and axo-axonic cells in the dentate gyrus

**DOI:** 10.3389/fnsyn.2025.1656759

**Published:** 2025-11-18

**Authors:** Rita M. Loureiro, Sam A. Booker, Akos Kulik, Imre Vida

**Affiliations:** 1 Institute for Integrative Neuroanatomy, Charité – Universitätsmedizin Berlin, Berlin, Germany; 2 Institute for Biology, Humboldt – Universität zu Berlin, Berlin, Germany; 3 Institute for Neuroscience and Cardiovascular Research, University of Edinburgh, Edinburgh, United Kingdom; 4 Simons Initiative for the Developing Brain, University of Edinburgh, Edinburgh, United Kingdom; 5 Patrick Wild Centre for Autism Research, University of Edinburgh, Edinburgh, United Kingdom; 6 Faculty of Medicine, Institute of Physiology II, University of Freiburg, Freiburg, Germany

**Keywords:** GABA_B_ receptors, excitatory plasticity, mossy fiber synapses, parvalbumin-expressing basket cells, axo-axonic cells, dentate gyrus

## Abstract

**Introduction:**

GABA_B_ receptors (GABA_B_Rs) are important modulators of neuronal excitability, synaptic transmission and plasticity in principal cells (PCs). While at the cellular level they can inhibit synaptic transmission directly, at the network level, due to a net disinhibitory effect, they promote plasticity in PCs. However, their effect on plasticity in GABAergic interneurons (INs) is less well-understood.

**Methods:**

In this study, we have combined quantitative immunoelectron microscopy and *ex vivo* whole-cell recordings to investigate the surface expression of GABA_B_Rs and their modulation of synaptic plasticity at mossy fiber (MF) inputs onto parvalbumin-expressing interneurons (PV-INs) in the rat dentate gyrus (DG).

**Results:**

Immunoelectron microscopy confirmed the expression of the GABA_B_Rs and their effector channel Kir3.1 on PV-IN dendritic shafts. Theta-burst extracellular stimulation of MFs resulted in robust long-term potentiation (LTP) in basket cells (BCs) and axo-axonic cells (AACs), the two main types of DG PV-INs. LTP in both types was strongly reduced, but not abolished, by the GABA_B_R agonist baclofen.

**Discussion/Conclusion:**

Finally, pre-application of SCH-23390, a blocker of Kir3 channels, occluded the inhibitory effect of baclofen on LTP. These results demonstrate that postsynaptic GABA_B_Rs negatively regulate synaptic plasticity at MF synapses onto DG perisomatic-inhibitory PV-INs via Kir3 channels.

## 1 Introduction

The dentate gyrus (DG) serves as the main input region to the hippocampus with pivotal roles in learning and memory, as well as spatial navigation (Borzello et al., 2023). As an interface between the entorhinal cortex (EC) and the CA3 area, the DG converts the rich cortical representation into a sparse hippocampal code. Diverse GABAergic interneurons (INs) play a particularly important role in this coding conversion by providing compartment-specific inhibition to dentate granule cells (DGCs). DG INs are recruited in the local circuit directly by EC afferents (feedforward inhibition) and by mossy fibers (MFs), the recurrent collaterals of DGCs (feedback inhibition). GABA released by DG INs acts via ionotropic GABA_A_ receptors (GABA_A_Rs) and metabotropic GABA_B_ receptors (GABA_B_Rs) to exert temporally and molecularly distinct forms of inhibition (Bettler et al., 2004; Ghit et al., 2021). While GABA_A_Rs localize predominantly to postsynaptic sites and mediate fast synaptic conductances, GABA_B_Rs are found on synaptic and extrasynaptic membrane on both pre- and postsynaptic microcompartments and regulate neuronal excitability and synaptic transmission on slower time scales (Kulik et al., 2018; Watson and Booker, 2024).

Beyond the direct cellular and synaptic inhibition, GABA_B_Rs play an important role as modulators of synaptic plasticity: in principal cells (PCs), GABA_B_R activation promotes long-term potentiation (LTP) (Davies et al., 1991; Mott and Lewis, 1991), despite direct, cellular level inhibitory effects (Davies and Collingridge, 1993). The facilitation of LTP by GABA_B_Rs results from inhibition of INs and thus GABA release, resulting in disinhibition in PCs (Davies et al., 1991; Mott and Lewis, 1991; Booker et al., 2020). In contrast, in a recent study we found that in somatostatin (SOM)-INs of the CA1 area GABA_B_R activation blocks synaptic plasticity at recurrent excitatory input synapses via inhibition of postsynaptic L-type Ca^2+^channels (Booker et al., 2018). These results support the hypothesis that, as a net effect, GABA_B_Rs can differentially affect plasticity in PCs and INs and change the long-term balance of excitation and inhibition in the network. However, in view of the high diversity of INs, it remains unknown whether negative modulation by GABA_B_R of synaptic plasticity in INs is a general principle with common molecular mechanisms.

Parvalbumin (PV)-expressing INs constitute a major subset of GABAergic INs, which provides strong and temporally-precise inhibition to the perisomatic domain of PCs as well as other INs in cortical circuits (Geiger et al., 1997; Bartos et al., 2001). PV-INs comprise two morphological types: basket cells (BCs) targeting the soma, and axo-axonic cells (AACs) innervating the axon initial segment (AIS) (Buhl et al., 1994; Degro et al., 2022). In the DG, PV BCs have been shown to express associative long-term synaptic plasticity at their recurrent (feedback) MF input on their basal dendrites in the hilus, but not at their perforant path (feedforward) synapses on their apical dendrites in the molecular layer (Alle et al., 2002; Sambandan et al., 2010). This plasticity at MF-BC synapses is not dependent on NMDA receptors (NMDARs), but involves Ca^2+^-permeable AMPA receptors (CP-AMPARs) (Alle et al., 2002; Sambandan et al., 2010). Whether GABA_B_R activation modulates this form of plasticity in DG BCs remains unknown. In this study, we tested the hypothesis that, similar to our observation in CA1 SOM-INs (Booker et al., 2018), GABA_B_Rs can negatively modulate LTP at feedback inputs to DG PV-INs. Additionally, we aimed to systematically identify AACs in order to assess whether synaptic plasticity and its modulation by GABA_B_Rs in this IN type shares properties with those in BCs.

## 2 Materials and methods

### 2.1 Experimental animals and details

A total of 57 transgenic Wistar rats (male and female, postnatal ages 18–30 days), expressing enhanced yellow fluorescence protein (Venus-YFP) under the vesicular GABA transporter (vGAT) promoter (Uematsu et al., 2008), were used for the electrophysiological recordings and slice immunostaining in this study. Samples for immunoelectron microscopy were obtained from existing material from 2 wild-type Wistar rats (6 weeks old) reported in a previous study (Booker et al., 2013). Sex-related differences were not checked. All experiments were performed in accordance with institutional (Charité – Universitätmedizin, Berlin), local (LaGeSo, Berlin, T 0215/11; LaGeSo, Berlin T-CH 0040/20; Freiburg G19/59), national guidelines (German Animal Welfare Act) and the European Council Directive 86/609/EEC.

### 2.2 Acute hippocampal slice preparation

Acute hippocampal slices were prepared from young rats as previously described (Booker et al., 2014). In brief, rats were deeply anaesthetised with isoflurane at 3% air saturation, decapitated, followed by swift removal of the brain and subsequently transferred into ice-cold, carbogenated (95% O_2_ / 5% CO_2_) sucrose-substituted artificial cerebrospinal fluid (sucrose aCSF; in mM: 87 NaCl, 2.5 KCl, 25 NaHCO_3_, 1.25 NaH_2_PO_4_, 25 glucose, 75 sucrose, 7 MgCl_2_, 0.5 CaCl_2_, 1 Na_2_-Pyruvate, 1 Na_2_-Ascorbate). The brain was sectioned into transverse slices of 300 μm thickness using a Vibratome (VT1200s; Leica, Wetzlar, Germany). Slices were placed in a sucrose aCSF-filled submerged holding chamber, incubated for 30 min at 34 °C and subsequently kept at room temperature.

### 2.3 Electrophysiological recordings

All electrophysiological recordings were performed in carbogenated aCSF (in mM: 125 NaCl, 2.5 KCl, 25 NaHCO_3_, 1.25 NaH_2_PO4, 25 glucose, 1 MgCl_2_, 2 CaCl_2_, 1 Na-Pyruvate, 1 Na-Ascorbate) in a submerged recording chamber filled with a constant flow of aCSF (4–6 mL/min) warmed to 33 ± 1 °C. Chemicals used in aCSF and sucrose aCSF were purchase from Sigma-Aldrich (St. Louis, MO) and Carl Roth (Karlsruhe, Germany).

Recording pipettes were made from borosilicate glass capillaries (Hilgenberg, Malsfeld, Germany) using a horizontal puller (P-97, Sutter Instruments, Novato, CA), with a series resistance of 3–6 MΩ when filled with biocytin-containing low-EGTA internal solution (in mM: 10 HEPES, 135 K-Gluconate, 10 KCl, 0.1 EGTA, 2 MgCl_2_, 2.69 Biocytin, 2 Na_2_ATP, 0.3 Na_2_GTP and 1 Na_2_Creatine). Stimulation electrodes were made from patch pipettes filled with 2 M NaCl.

An upright microscope (Scientifica, Uckfield, UK) equipped with infra-red oblique illumination and epifluorescence illumination with an YFP filter set (F36-528, AHF, Tübingen-Pfrondorf, Germany) and LED excitation (499 nm, CoolLED, Andover, UK), and a digital camera (Q Imaging, Surrey, BC) were used to identify and target YFP-positive GABAergic cells.

Voltage- and current-clamp recordings were performed using an Axon 700B amplifier (Molecular Devices, San Jose, CA), filtered at 10 kHz using the built-in low-pass Bessel filter, and digitised at a sampling frequency of 20 kHz using an USB-6259 AD-converter interface (National Instruments, Austin, TX). Data was recorded using WinWCP (courtesy of John Dempster, Strathclyde University, Glasgow, UK^1^) and later analysed using the Stimfit open-source package (Guzman et al., 2014).^2^

All cells were characterized in current-clamp mode at resting membrane potential. A set of hyper- and depolarising current steps (from −250 pA to 600 pA, steps of 50 pA, 500 ms duration) were applied to identify the cells based on their firing pattern. In all current-clamp experiments bridge balance and pipette capacitance were fully compensated.

Recordings were discarded or excluded from analysis if membrane potential of the recorded neuron was more depolarized than −50 mV, initial series resistance exceeded 30 MΩ or instability in the recording was detected when accompanied by change of series resistance by >20% during the experiment.

### 2.4 Analysis of GABA_B_R-mediated currents in PV-INs

To isolate GABA_B_R-mediated currents, AMPAR antagonist DNQX or CNQX (10 μM; Abcam, Cambridge, UK; or Ascent, Bristol, UK; respectively), the NMDAR antagonist D-AP5 (50 μM; Abcam), and the GABA_A_R antagonist SR95531 (Gabazine; 10 μM; Abcam) were added to the bath. Cells were recorded in voltage-clamp mode and held at −65 mV.

Whole-cell currents were induced pharmacologically by adding the selective GABA_B_R agonist (RS-) baclofen (10 μM; TOCRIS, Bristol, UK) to the bath. To test for selectivity of the induced whole-cell current, baclofen was subsequently washed out and the selective antagonist, CGP-55845 hydrochloride (CGP; 5 μM; TOCRIS) applied.

To test the involvement of Kir3 channels, we preapplied the Kir3 channel blocker SCH-23390 (SCH; 10 μM; TOCRIS) to the aCSF. Baclofen was co-applied after about 10 min.

GABA_B_R-mediated effects were measured as the difference between the whole-cell current measured during the steady state plateau during wash-in and the baseline directly preceding the drug application (averages over 5 min periods). Additionally, pharmacologically-isolated GABA_B_R-mediated inhibitory postsynaptic currents (IPSCs) were elicited via extracellular stimulation (5 pulses at 200 Hz, Booker et al., 2013) to the outer molecular layer of the DG. IPSC amplitude change was calculated as a percentage of the change after influx of baclofen normalized to the baseline.

### 2.5 Assessment of synaptic plasticity in PV-INs

To stimulate MF input to PV-INs, the extracellular stimulation electrode was positioned in the hilus of the DG, approximately 100 μm away from the recorded cell. Excitatory postsynaptic potentials (EPSPs) were evoked by single current pulses every 10 s. First, an input/output (I/O) curve was explored by increasing stimulus intensity at fixed increments until the recorded cell produced an action potential (AP) or the EPSP amplitude reached plateau. For test pulses the intensity was reduced to 30–50% of the determined maximum. EPSPs were collected for at least 5 min to achieve a stable baseline. To induce plasticity, a theta-burst stimulus (TBS) protocol was applied (Alle et al., 2002; Sambandan et al., 2010). The TBS consisted of 12 bursts of stimuli delivered at a frequency of 0.3 Hz; each burst was composed of 25 stimuli applied at a frequency of 30 Hz (Alle et al., 2002; Sambandan et al., 2010). Each stimulus was paired with a brief suprathreshold depolarising current pulse (0.2 ms duration, 2 ms delay). The TBS was delivered 3 times with 30 s intervals.

EPSP amplitudes were normalized to 5 min baseline immediately preceding the TBS and expressed as percentage. Post-tetanic potentiation (PTP) was taken as the amplitude of the first recorded stimulus immediately after the induction protocol was delivered, excluding APs. LTP was assessed between 25 to 30 min after the induction protocol. In a subset of control experiments, DCG-IV (DCG; 5 μM; TOCRIS) was applied to the slices either at the end of the LTP recording (>30 min after the TBS, 2 cells) or in slices with baseline recording only (2 cells) to confirm that they were MF mediated (Alle et al., 2002).

To assess the effect of GABA_B_Rs, baclofen (10 μM) and CGP (5 μM) were pre-applied (>20 min) to the bath and plasticity was assessed as above under the influence of these drugs. Additional LTP experiments were performed in the presence of SCH (10 μM, preapplied to the bath) with baclofen co-application.

### 2.6 Visualization of the recorded neurons and immunocytochemistry

Following successful recording, the pipettes were cautiously withdrawn to allow the neurons to reseal. Slices were immediately immersion fixed in phosphate buffer (PB, 0.1 M) based solution containing 4% paraformaldehyde (PFA) overnight at 4 °C. Slices were then transferred to and stored in 0.1 M PB. Slices were processed using a protocol previously described by our lab (Bolduan et al., 2020). Slices were washed thoroughly in PBS (0.1 M PB + 0.9% NaCl) and incubated in a blocking solution (PBS, 0.5% Triton X, 10% Normal Goat Serum (NGS), 0.05% Sodium Azide) for 1 h, prior to being incubated at 4 °C over 2 days in a primary-antibody solution (PBS, 0.3% Triton X, 5% Normal Goat Serum (NGS), 0.05% Sodium Azide) containing primary antibody against PV (mouse monoclonal, dilution: 1:5000; Swant, Burgdorf, Switzerland). A subset of slices were also incubated with a primary antibody against Ankyrin G (rabbit polyclonal, dilution: 1:1000; Santa Cruz Biotechnology, Dallas, TX) to visualize AIS. After the incubation period, slices were repeatedly washed in PBS and incubated overnight at 4 °C with an AlexaFluor 405-conjugated secondary-antibody (goat anti-mouse, dilution: 1:500; Invitrogen, Waltham, MA) in PBS with 0.1% Triton X, 3% NGS, and 0.05% Sodium Azide. To visualize the recorded, biocytin-filled neurons, AlexaFluor 647-conjugated streptavidin (dilution: 1:500; Invitrogen) was added to this solution. Slices were repeatedly washed in 0.1 M PB, before mounting them onto standard slides with 300 μm thick metal spacers (Bolduan et al., 2020) and embedded in an aqueous mounting medium (Fluoromount-G, Southern Biotech, AL, USA). All slices were kept in storage at 4 °C.

### 2.7 Imaging and reconstruction

A laser scanning confocal microscope (FluoView 1000, Olympus, Tokyo, Japan) was used to image the biocytin-filled cells using a x30 objective (silicon oil-immersion, 1.05 N.A., 0.8 mm W.D., Olympus), or a x60 objective (silicon oil-immersion, 1.35 N.A., 0.3 mm W.D., Olympus). Morphological identification of PV-INs was performed using ImageJ software (Bethesda, MD).^3^ PV-expressing perisomatic-inhibitory INs were identified (1) by their immunoreactivity for PV or (2) by their characteristic axonal distribution within and near the GCL. BCs and AACs were further classified based on their characteristic axonal distributions: (1) nest-like ramifications around somata and proximal dendrites of DGCs in and above the GCL (BCs), or (2) cartridge-like vertical collaterals within and below the GCL (AACs), respectively. Neurons were classified by three independent observers. Neurons that were differentially classified were all included in the subset of slices immunstained for Ankyrin-G to visualize the AIS and to evaluate the apposition of their axon terminals. A subset of the neurons were fully reconstructed for illustration purposes using Neutube software (Janelia Research Campus, Ashburn, VA).^4^

### 2.8 Pre-embedding immunoelectron microscopy

Samples for electron microscopic analysis were obtained from existing immunostained material from which the subcellular localization of GABA_B_R and Kir3 channel subunits were previously characterized in CA1 PV-INs and PCs (Booker et al., 2013).

In brief, the material was obtained from 2 adult male Wistar rats. The animals were first sedated with isoflurane and then terminally anesthetized with pentobarbital (50 mg/kg, intraperitoneally) and transcardially perfused with 0.9% NaCl for 1 min followed by a fixative solution containing 4% paraformaldehyde (Merck, Darmstadt, Germany), 0.05% glutaraldehye (Polyscience, Warrington, PA) and 15% (v/v) saturated picric acid (Sigma-Aldrich) in 0.1 M PB for 12 min.

For immunolabeling, hippocampal sections were cut on a vibratome (VT 1000, Leica) at a thickness of 60 μm, freeze/thaw permeabilized, then blocked. Affinity-purified and characterized polyclonal rabbit antibodies were used to detect the GABA_B1_ subunit (B17, Kulik et al., 2002), as well as the Kir3.1 and Kir3.2 subunits (Alomone Laboratories, Jerusalem, Israel). PV-expressing INs were identified using the mouse monoclonal antibody (Swant). The sections were incubated with primary antibodies (GABA_B1_: 2 μg/mL, Kir3.1: 1.5 μg/mL, Kir3.2: 2 μg/mL in combination with PV 1:8000) diluted in Tris-buffered saline (TBS) containing 3% NGS (Millipore, Burlington, MA) overnight. Sections were then washed and transferred into a mixture of secondary antibodies: goat-anti rabbit (Fab fragment, 1:100) coupled to 1.4 nm gold particle (Nanoprobes, Stony Brooks, NY) and biotinylated goat anti-mouse antibodies (1:50, Vector Laboratories, Burlingame, CA). After repeated washes in TBS, sections were washed in double-distilled water, followed by silver enhancement of the gold particles with an HQ Silver kit (Nanoprobes). Subsequently, the sections were incubated with avidin-biotin peroxidase complex (ABC kit, Vector Laboratories) made up in TBS and washed in Tris buffer (TB). Peroxidase was visualized with 3,3′-diaminobenzidine tetrahydrochloride (DAB, 0.05% in TB) as a chromogen and 0.01% H_2_O_2_ as substrate. The sections were treated with 1% OsO_4_ in PB for 40 min and 1% uranyl acetate for 35 min. They were dehydrated in a series of ethanol and propylene oxide and flat embedded in epoxy resin (Durcupan ACM Fluka, Sigma-Aldrich). Serial ultrathin sections from the hilus of the DG were cut at 80 nm thickness from the very surface of the section (up to 3 μm in depth, Kulik et al., 2002) using an ultramicrotome (Reichert Ultracut E, Leica, Austria) then they were imaged and analyzed using a JEM 2100 Plus electron microscope (Jeol, Japan).

PV-positive dendritic shafts, indicated by the homogeneous intracellular peroxidase reaction end-product, were analyzed in consecutive images. Putative excitatory synapses on PV-labeled dendritic shafts were identified by their asymmetrical appearance. Immunogold density was calculated by dividing the number of immunogold particles on the inner leaflet of the plasma membrane and the surface area of dendritic profiles. The surface area of plasma membrane was determined by measuring the perimeter of dendrites (ImageJ software) and multiplying it by the nominal ultrathin section thickness.

### 2.9 Statistical analysis

Statistical analyses were performed using Prism 8 (GraphPad; San Diego, CA) software packages. Changes in the EPSP amplitude (e.g., during PTP, LTP) were normalized to baseline and tested using paired *t*-test. We used D’Agostino & Pearson test to assess whether LTP data were normally distributed. Based on those results, we used Kruskal-Wallis test and Dunn’s post-hoc test to determine the pharmacological effects on LTP where multiple groups were compared (control vs. baclofen vs. CGP). Pharmacological effects on LTP and PTP in BCs and AACs and in the presence of SCH, changes in EPSP amplitude following application of DCG and baclofen, and changes in whole-cell currents were tested by the Mann–Whitney test. Statistical significance was accepted at *p* < 0.05 and results were presented as mean ± SEM unless indicated otherwise. Abbreviations used: n.s. – Not Significant.

## 3 Results

To confirm the presence of functional GABA_B_Rs and to determine their contribution to the modulation of synaptic plasticity, we performed whole-cell recordings from PV-INs in rat acute hippocampal slices obtained from rats expressing Venus fluorescent protein (YFP) under the vesicular GABA transporter (vGAT) promoter (Uematsu et al., 2008).

**Figure 1:**
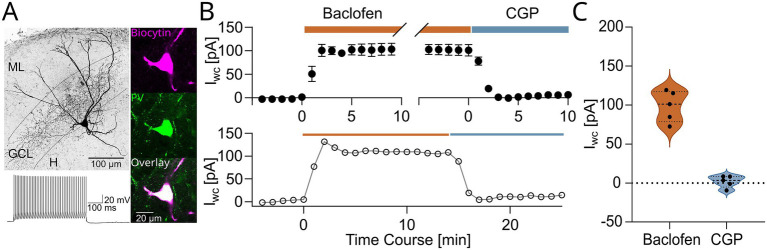
Postsynaptic GABA_B_R-mediated currents in DG fast-spiking perisomatic-inhibitory PV-INs. **(A)** A projected image from confocal stacks (inverted gray scale) shows a recorded and biocytin-filled PV+ IN. Note the extensive axonal arborisation in the granule cell layer (GCL). Inset to the right illustrates the visualized biocytin-filled cell body (magenta, top), the PV immunolabeling (green, middle), and the overlay (bottom). Inset on the bottom shows the high frequency, non-accommodating discharge of the IN in response to a large depolarizing current pulse (600 pA, 500 ms). **(B)** Upper panel: Time course plot of whole-cell currents (I_WC_) induced by baclofen (wash-in: orange bar) and reversed by subsequent application of CGP (blue bar). Lower panel: Representative time course plot of I_WC_ from a single cell. **(C)** Summary plot of the changes in steady state whole-cell currents measured during baclofen wash-in (orange) and subsequent CGP application (blue). Violin plots show median as solid line and upper and lower quartiles as dotted lines.

PV-INs were selected for recording based on (i) localization of somata near the granule cell layer (GCL)/hilus border and (ii) YFP fluorescence. Their identity was first established based on their fast-spiking (FS) discharge pattern in response to depolarizing pulses during the recordings (Figure 1A, inset). Recorded INs were filled with biocytin for subsequent visualization to enable immunohistochemical and morphological characterizations (Figure 1A). Based on the inclusion criteria (see Methods), we included 62 recorded neurons as FS, perisomatic-inhibitory PV-INs, 59 of which were morphologically confirmed as having perisomatic axons. PV immunolabeling was confirmed in 49 of the 62 cells, including the 3 cells lacking axon.

Inspection of the axonal distribution of PV-INs further revealed the presence of the two distinct PV-IN populations: (i) BCs (39 INs) characterized by nest-like ramifications around somata of DGCs somata, and (ii) AACs (12 INs) identified by their cartridge-like vertical collaterals within and below the GCL, where the axon initial segments of DGCs are distributed (Buhl et al., 1994; Degro et al., 2022). In the remaining PV-INs (*n* = 8), while axon collaterals were observed in the cell body layer, identifying them as perisomatic INs, unequivocal classification as BC or AAC was not possible due to the limited extent of these collaterals.

### 3.1 Functional expression of GABA_B_Rs and Kir3 channels in PV-INs of the DG

We have recently demonstrated the presence of postsynaptic GABA_B_R-mediated currents in PV-INs in CA1 (Booker et al., 2013) and DG (Degro et al., 2024). To confirm this finding for our experimental conditions, we first applied the GABA_B_R agonist baclofen (10 μM) during whole-cell recordings from a subset of PV-INs (5 PV-INs, from 5 animals). From −65 mV, baclofen induced outward whole-cell currents with a mean amplitude of 98.6 ± 8.9 pA (Figures 1B,C). Wash-out of baclofen combined with the application of the selective GABA_B_R antagonist CGP-55,845 (CGP; 5 μM) resulted in reversal of this outward current (mean amplitude from preceding baseline: 1.9 ± 3.3 pA, 5 PV-INs, from 5 animals, *p* = 0.0001, *t* = 14.77, paired *t*-test; Figures 1B,C), confirming the receptor specific nature of the baclofen-induced current.

**Figure 2:**
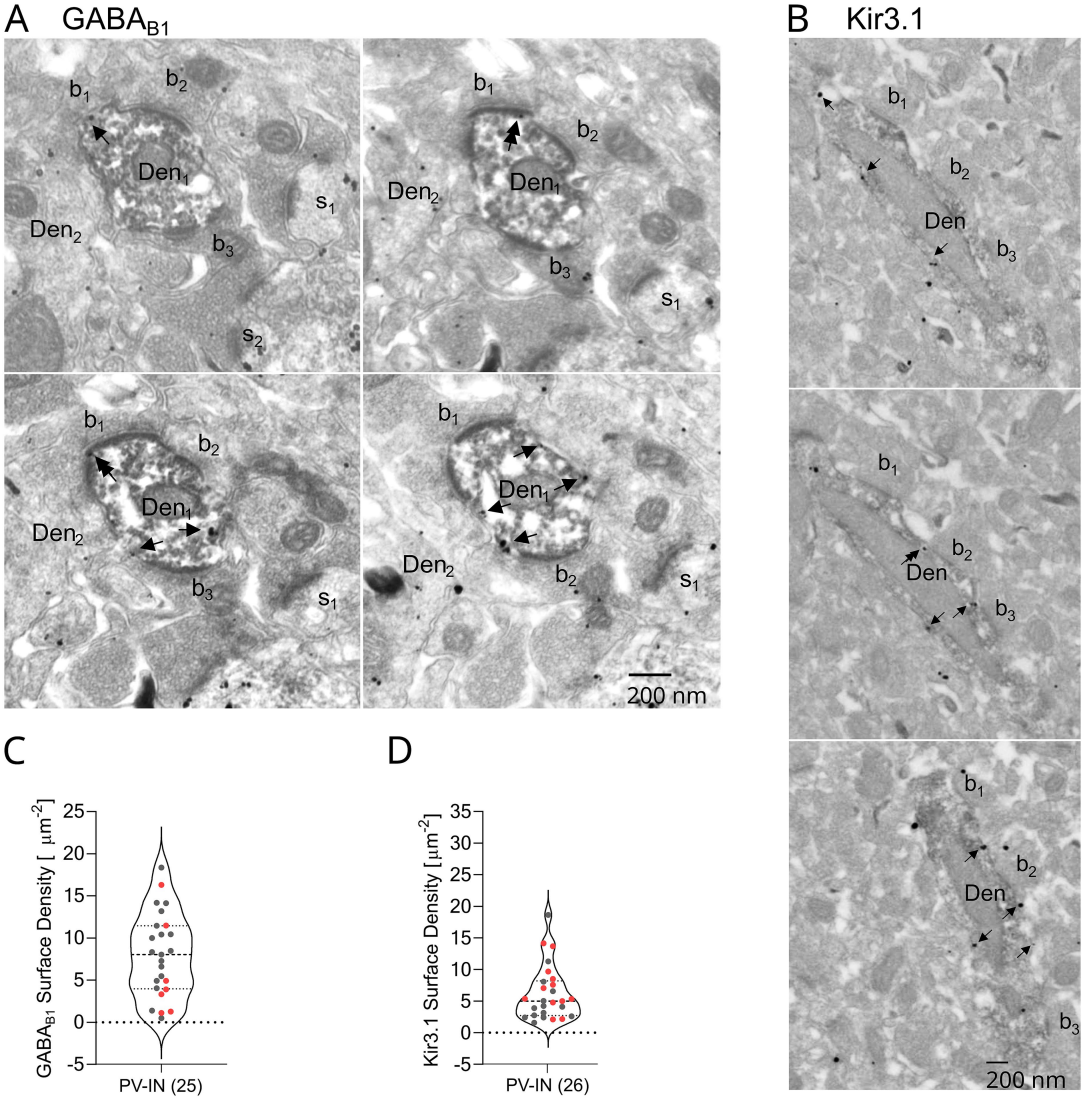
GABA_B_Rs and Kir3 channels are expressed on the basal dendrites of PV-INs. **(A)** Consecutive electron micrographs show the localization of immunogold particles for the GABA_B1_ subunit to extrasynaptic (arrows) and perisynaptic positions (double arrows) over the membrane surface of PV+ dendrites (Den_1_, peroxidase reaction end-product) in the DG hilus. Axon terminals (b_1_–b_3_) of putative excitatory cells contact Den_1_. Note the immunoreactivity for the receptor subunit was also detected in a neighbouring dendritic shaft (Den_2_) and spines (s_1_–s_2_) of peroxidase-negative neurons. **(B)** Consecutive electron micrographs illustrate immunoreactivity for the Kir3.1 subunit (immunogold particles) in extrasynaptic (arrows) and perisynaptic positions (double arrow) on the membrane of a PV+ dendrite shaft (Den, peroxidase reaction end-product) contacted by axon terminals (b_1_–b_3_) of putative excitatory cells. **(C)** Summary plot of the surface density of GABA_B1_ particles on PV+ dendrites (7.9 ± 0.9 particles/μm^2^). PV-IN data collected from two animals color-coded as animal 1 (gray dots) and animal 2 (red dots). **(D)** Summary plot of the surface density of Kir3.1 particles on PV+ dendrites (6.1 ± 0.8 particles/μm^2^). PV-IN data collected from two animals color-coded as animal 1 (gray dots) and animal 2 (red dots). Number of analyzed dendrites are in parentheses. Scale bars, 200 nm. All violin plots show median as solid line and upper and lower quartiles as dotted lines.

To confirm the surface localization of GABA_B_Rs and their effector Kir3 channels to the dendrites of PV-INs, we next performed electron microscopic (EM) analysis of pre-embedding immunogold labeling for the GABA_B1_ and Kir3.1 subunits in combination with immunoperoxidase labeling for PV in tissue samples from the hilus. Immunogold particles for GABA_B1_ were mainly found along the extrasynaptic plasma membrane of immunoperoxidase-labeled PV-expressing (PV+) dendritic shafts (Figure 2A). Occasionally, they also appeared at the edge of asymmetric synapses established by axon terminals of putative excitatory cells with dendritic shafts (Figure 2A). Quantification of the surface density of immunoparticles for GABA_B1_ on PV + dendrites in serial ultrathin sections revealed a mean immunogold density of 7.9 ± 0.9 particles/μm^2^ (*n* = 25 dendrites, from 2 animals; Figure 2C). The variability among the sampled dendrites was high, ranging from 0.5 particles/μm^2^ to 18.3 particles/μm^2^.

Particles for Kir3.1, similar to the receptor subunit, were also found to predominantly localize to the extrasynaptic membrane of immunoperoxidase-labeled PV-IN dendrites and, to a lesser extent, to perisynaptic membrane of putative glutamatergic synapses (Figure 2B). The surface density of immunoparticles for Kir3.1 with a mean of 6.1 ± 0.8 particles/μm^2^ was comparable to that of GABA_B1_, and also showed high variability, ranging from 1.6 particles/μm^2^ to 18.6 particles/μm^2^ (*n* = 26 dendrites, from 2 animals; Figure 2D). The surface density of both GABA_B1_ and Kir3.1 was lower on DG PV-INs than what was observed on the dendrites of CA1 PV-INs and PCs in our previous study (Booker et al., 2013; Figure 2C there). In contrast to Kir3.1, no discernible immunolabeling for the Kir3.2 subunit was observed on dendritic shafts of PV + neurons (not shown). Together, these data confirm the surface localization of both GABA_B_Rs and Kir3 channels in PV-INs.

### 3.2 GABA_B_R activation diminishes LTP at the excitatory synapses of DG PV-INs

**Figure 3:**
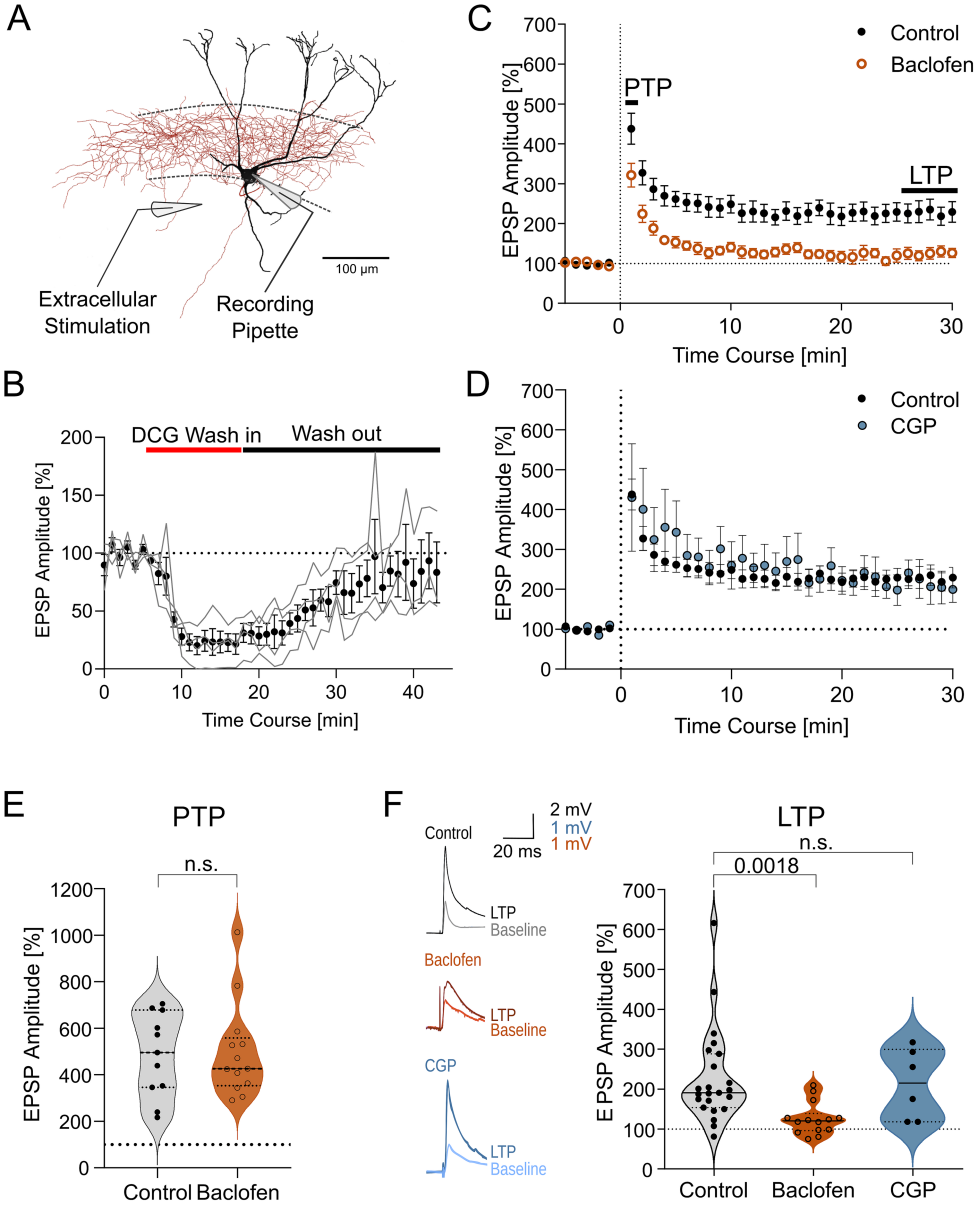
Activation of GABA_B_Rs suppresses LTP on PV-INs. **(A)** Schematic of the experimental configuration showing a recording from a basket cell (reconstruction of the IN illustrated in Figure 1A) and an extracellular stimulating electrode placed in the hilus of the DG to stimulate the MF input to the neuron. **(B)** Time course plot of EPSP amplitude (normalized to preceding baseline) during application of the group II metabotropic glutamate receptor agonist DCG-IV and after wash-out (gray area) from 4 PV-INs. Red bar shows the approximate average DCG influx period and black bar shows the approximate average wash out period. Individual experiment traces showed in gray lines. **(C)** Time course plot of EPSP amplitudes (binned per minute) before and after theta-burst LTP induction protocol was applied in control conditions (23 PV-INs, black filled circles) and in the presence of baclofen (14 PV-INs, orange open circles). **(D)** Time course plot of EPSP amplitudes during a similar LTP experiment in control conditions (23 PV-INs, black filled circles) and in the presence of CGP (6 PV-INs, blue filled circles). **(E)** Summary plot of the mean EPSP amplitudes directly after the induction protocol (post-tetanic potentiation, PTP) in control condition (11 PV-INs; in gray) and after baclofen application (13 PV-INs; in orange). **(F)** Summary plot of the mean EPSP amplitudes between 25–30 min after the induction protocol (LTP) in control conditions (24 PV-INs), in baclofen (14 PV-INs) and in CGP (6 PV-INs). *Insets to the left*, representative traces of EPSPs (averages of 10 individual traces) acquired directly before (Baseline; thin lines) and 25–30 min after induction protocol in PV-INs (LTP; thick lines) in the three different conditions, control in gray, baclofen in orange, and CGP in blue. Violin plots show median as solid line and upper and lower quartiles as dotted lines.

To determine the role that GABA_B_R activation plays on synaptic plasticity at excitatory feedback inputs to PV-INs, we stimulated MF inputs via an extracellular electrode placed in the hilus, approximately 100 μm away from the recorded IN somata (Figure 3A). In a subset of experiments we confirmed that our stimulation paradigm selectively recruited the MF pathway by bath-applying DCG-IV (5 μM), a group II metabotropic glutamate receptor agonist. DCG-IV resulted in a marked reduction in the amplitude of the evoked EPSPs to 23 ± 9% in steady-state from baseline (4 PV-INs, from 4 animals, *p* = 0.03, U = 0, Mann–Whitney test; Figure 3B). Wash-out of DCG-IV resulted in a substantial recovery of EPSP amplitude to baseline level (100 ± 16%, 4 PV-INs, from 4 animals, *p* = 0.99, U = 8, Mann–Whitney test).

LTP was induced by applying three trains of 25 pulses at 30 Hz, separated by 30 s intervals (Sambandan et al., 2010; Figure 3A, inset). Immediately following induction protocol, the EPSP amplitude markedly increased, corresponding to a post-tetanic potentiation (PTP) (Alle et al., 2002). The increase of the EPSP amplitude was 485 ± 54% from a mean baseline level of 3.8 ± 0.4 mV (11 PV-INs, from 10 animals, *p* < 0.0001, *t* = 7.143, paired *t*-test; Figures 3C,E). In a subset of the neurons (12 PV-INs), the PTP resulted in action potential generation and the amplitude could not be measured precisely. PTP rapidly declined (mean decay time constant: 3.7 ± 1.1 min) and the EPSP amplitudes subsequently stabilized, and remained elevated for the following 30 min of the recording. Mean amplitude of EPSPs was 227 ± 25% of baseline, measured at 25–30 min after the induction (23 PV-INs, from 22 animals, *p* < 0.0001, *t* = 5.171, paired *t*-test; Figures 3C,F), confirming the expression of an LTP.

In the presence of the bath-applied GABA_B_R agonist baclofen (10 μM), the induction protocol similarly resulted in an initial PTP with a mean increase of 498 ± 57% of the baseline EPSP amplitude of 1.6 ± 0.2 mV (13 PV-INs, from 13 animals, *p* < 0.0001, *t* = 7.044, paired *t*-test; Figures 3C,E). The magnitude of the PTP was comparable to the one produced in baclofen-free conditions (11 PV-INs, from 10 animals, *p* = 0.86, U = 68, Mann–Whitney test). Following the PTP, EPSP amplitude stabilized at level above baseline. The mean EPSP amplitude measured at 25–30 min after induction was 126 ± 11% (14 PV-INs, from 14 animals, *p* = 0.03 compared to baseline, *t* = 2.359, paired *t*-test; Figures 3C,F), indicating an LTP. However, the magnitude of the LTP was substantially lower than that produced in baclofen-free condition (*p* = 0.003, Kruskal-Wallis test = 11.92, with Dunn’s post-test: *p* = 0.002; Figure 3F).

Our results, thus, suggest that GABA_B_Rs do not abolish but act as a negative modulator of synaptic plasticity. To test if antagonizing GABA_B_Rs affects the induction of LTP, we bath-applied CGP (5 μM) and induced plasticity in a set of PV-INs. We observed that blocking GABA_B_Rs did not further potentiate LTP induction (213 ± 36%, 6 PV-INs, from 4 animals, *p* = 0.003, Kruskal-Wallis test = 11.92, with Dunn’s post-test: *p* > 0.99; Figures 3D,F), suggesting that GABA_B_Rs do not tonically inhibit plasticity in our slice conditions.

### 3.3 Comparison of LTP and its suppression by baclofen in BCs and AACs

**Figure 4:**
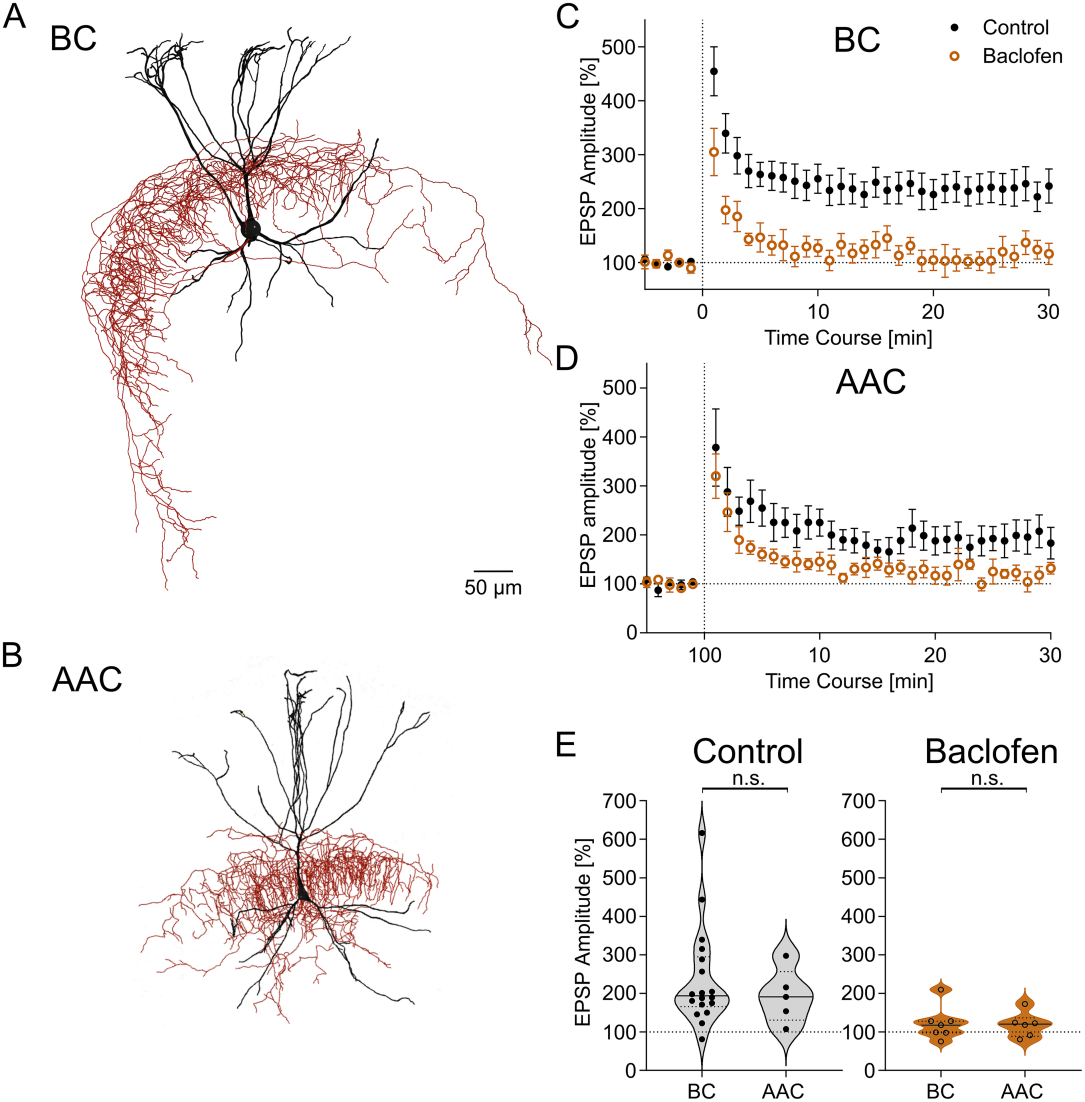
LTP induction and its modulation by GABA_B_Rs are similar in PV BCs and AACs. **(A, B)** Reconstruction of a basket cell (BC) and axo-axonic cell (AAC) from the DG. **(C, D)** Time course plots of EPSP amplitudes (binned per minute) before and after theta-burst LTP induction protocol was applied in control conditions (18 BCs, 5 AACs; black filled circles) and in the presence of baclofen (7 BCs, 6 AACs; orange open circles) for BCs and AACs. **(E)** Summary plots of mean EPSP amplitudes between 25–30 min after the induction protocol (LTP) in control conditions (*left plot*) for confirmed BCs and AACs and in the presence of baclofen (*right plot*). Violin plots show median as solid line and upper and lower quartiles as dotted lines.

As our sample of DG PV-INs comprised both BCs and AACs (Figures 4A,B), we analyzed our data to compare these two morphologically distinct IN types. Under control conditions, LTP was successfully induced in both BCs (237 ± 30% of baseline, 18 BCs, from 17 animals, *p* = 0.0005, *t* = 4.379, paired *t*-test; Figures 4C–E) and AACs (193 ± 32% of baseline, 5 AACs, from 5 animals, *p* = 0.04, *t* = 2.920, paired *t*-test; Figures 4D,E) and the change in EPSP amplitudes was not significantly different in the two PV-IN types (*p* = 0.75, U = 40, Mann–Whitney test; Figure 4E). When baclofen was pre-applied, the level of LTP was substantially reduced in both BCs (122 ± 16%, 7 BCs, from 7 animals, *p* = 0.22, *t* = 1.362, paired *t*-test; Figures 4C–E) and AACs (118 ± 13%, 6 AACs, from 6 animals, *p* = 0.23, *t* = 1.384, paired t-test; Figures 4D,E). The level of LTP in this condition was comparable between the two IN types (*p* = 0.95, U = 20, Mann–Whitney test; Figure 4E).

These data provide evidence that BCs and AACs express similar levels of LTP at their MF input synapses and the negative modulatory effect of GABA_B_R activation is also comparable.

### 3.4 GABA_B_Rs modulate synaptic plasticity by activating postsynaptic Kir3 channels in PV-INs

**Figure 5:**
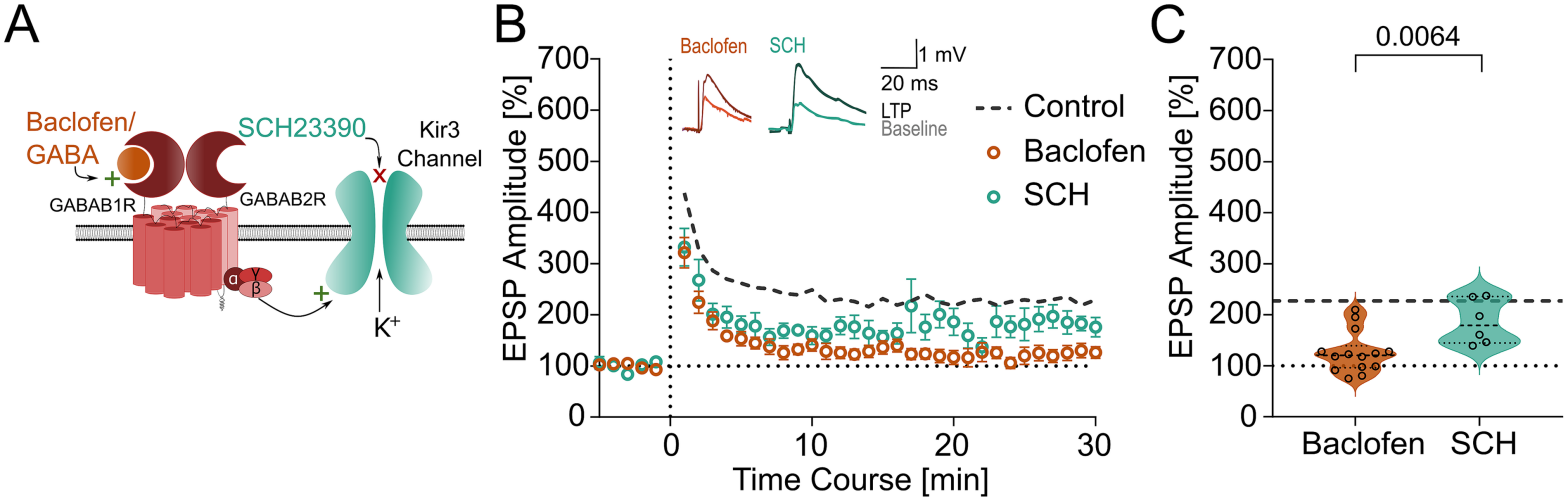
Postsynaptic GABA_B_Rs modulate synaptic plasticity by activating Kir3 channels. **(A)** Schematic of targets of baclofen and SCH on the GABA_B_R-Kir3 molecular complex. Baclofen is an agonist of GABA_B_Rs, acting at GABA_B1_R subunit that leads to activation of the Kir3 channel causing it to open and allowing outflow of K^+^ ions. SCH targets directly the Kir3 channel to prevent its opening. **(B)** Time course plot of EPSP amplitudes (binned per minute) before and after theta-burst LTP induction protocol was applied in baclofen conditions (14 PV-INs, orange open circles) and in the presence of SCH co-applied with baclofen (6 PV-INs, green open circles). Dashed line represents the mean level of LTP in control conditions (23 PV-INs). *Insets,* representative EPSPs (averages of 10 individual traces) acquired before (Baseline; thin lines) and 25–30 min after induction (LTP; thick lines) in the presence of baclofen (left) or SCH (right). **(C)** Summary plot of the mean EPSP amplitudes between 25–30 min after the induction protocol (LTP) in baclofen (14 PV-INs) and SCH (6 PV-INs) conditions. Violin plots show median as solid line and upper and lower quartiles as dotted lines.

The divergent effect of GABA_B_R activation on LTP compared to PTP suggests that the modulatory effect of baclofen is likely to be postsynaptic, given that PTP is largely presynaptic (Alle et al., 2002). To test whether the canonical postsynaptic effector Kir3 channel-mediated signaling is involved in the modulation of LTP, we next applied the channel blocker SCH-23390 (SCH, 10 μM; Figure 5A) to the bath and repeated the plasticity experiments in the presence and absence of baclofen.

Wash in of SCH itself did not affect the baseline whole-cell current in PV-INs (−15.9 ± 6.7 pA, 4 PV-INs, from 3 animals). Subsequent bath application of baclofen in the presence of SCH resulted in an outward whole-cell current (33.7 ± 7.1 pA), which was significantly lower than the whole-cell current induced in the absence of SCH (see above, Figure 1B, *p* = 0.006, U = 0, Mann–Whitney test). Additionally, we induced GABA_B_R-mediated slow IPSCs and analyzed the change in IPSC amplitude upon application of baclofen in control conditions and in the presence of SCH. We observed a similar level of reduction in the slow IPSC in the two conditions (control: 45.9 ± 10.0%, 4 PV-INs, from 4 animals; SCH: 46.6 ± 10.1%, 4 PV-INs, from 3 animals; *p* = 0.89, U = 7, Mann–Whitney test). These results confirm that postsynaptic GABA_B_R activation is largely mediated by SCH-sensitive Kir3 channels.

When SCH was pre-applied to the bath, LTP was readily induced in the presence of baclofen (EPSP amplitudes 186 ± 18% of the baseline mean EPSP amplitude of 2.0 ± 0.6 mV, 6 PV-INs, from 6 animals, *p* = 0.005, *t* = 4.810, paired *t*-test; Figures 5B,C). LTP was significantly larger in this condition than the one induced in the presence of baclofen (*p* = 0.006, U = 10, Mann–Whitney test). Furthermore, the LTP was not significantly different when compared to the control LTP induced in the absence of baclofen (*p* = 0.55, U = 57, Mann–Whitney test).

These results, thus, indicate that GABA_B_R negatively modulated plasticity at the MF—PV-IN synapses is postsynaptic and involves the Kir3 channel-mediated signaling.

## 4 Discussion

In this study, we demonstrate that functional postsynaptic GABA_B_Rs and their effector Kir3 channels are expressed on the basal dendrites of PV-INs in the DG hilus. Activation of these receptors results in suppression of LTP induction at the synapses formed by MFs onto the basal dendrites of the PV-INs. The suppressive effect of GABA_B_Rs on synaptic plasticity is postsynaptic and involve Kir3 channels. Together, the data suggest that GABA_B_Rs not only reduce excitability of neurons and synaptic transmission, with a net disinhibition to the DG network (Mott et al., 1993; Foster et al., 2013), but can also tilt the long-term balance of excitation and inhibition by negatively modulating the recruitment of feedback inhibition to DGCs via PV-INs.

### 4.1 Expression of GABA_B_ receptors and Kir3 channels in PV-INs

GABA_B_Rs have been shown to be widely expressed in both PCs and INs of the hippocampus (Kulik et al., 2003). Despite early light microscopic studies found no evidence for the presence of GABA_B_Rs in PV-INs (Sloviter et al., 1999; Freund and Katona, 2007), we previously demonstrated that in the CA1 area PV-BCs express both GABA_B_R subunits, GABA_B1_ and GABA_B2_, in their postsynaptic compartments (Booker et al., 2013). In the DG, prior electrophysiological data have shown that GABA_B_R activation produces outward current and slow inhibitory postsynaptic potentials (IPSPs) in PV-BCs (Mott et al., 1999; Degro et al., 2024). Results of our study confirm the presence of GABA_B_R-mediated currents in DG PV-INs and also provide direct evidence for the surface localization of GABA_B_R subunit 1 on basal dendrites of these INs. In comparison to both PV-INs and PCs of the CA1, however, the average surface density is substantially lower in DG PV-INs (~40%, 7.9 ± 0.9 versus 13.0 ± 1.7 particles/μm^2^, Booker et al., 2013).

We further observed immunolabeling for the postsynaptic effector channel subunit Kir3.1 on the membrane surface of DG PV-IN dendrites, but not for Kir3.2. This is in contrast to our previous result from CA1 area demonstrating consistent presence of both Kir3.1 and 3.2 subunits on the dendritic surface of PV-INs (Booker et al., 2013). The reason for the lack of Kir3.2 is particularly intriguing, as functional Kir3 channels are believed to be mainly composed of the Kir3.1 and Kir3.2 subunits in cortical regions (Lesage et al., 1995; Leaney, 2003). In fact, Kir3.2 has been proposed to be essential for assembly and surface localization of the channels (Inanobe et al., 1999; Ma et al., 2002). However, there is evidence that Kir3.1 subunits may form functional homotetramers (Corey and Clapham, 1998) and, they may also combine with Kir3.4 to form heteromultimeric channels (Krapivinsky et al., 1995; Dascal, 1997). The finding here, thus, may reflect an unusual composition of the effector Kir channels in DG PV-INs, but this aspect requires further investigations.

### 4.2 GABA_B_ receptor activation suppresses the induction of synaptic plasticity in DG PV-INs

LTP at the excitatory input from DGC axons, the MFs, onto the basal dendrites of PV-INs is a well documented form of IN plasticity, important for the modulation of the recruitment of feedback inhibition onto DGCs (Alle et al., 2002; Sambandan et al., 2010; Hainmüller et al., 2014). Among the diverse forms of plasticity observed in the various IN types (Kullmann and Lamsa, 2007; Bartos et al., 2011), this associative LTP phenomenologically resembles Hebbian plasticity observed in PCs, as its induction requires precise timing of synaptic activation and postsynaptic AP generation, as well as postsynaptic calcium signaling (Alle et al., 2002; Sambandan et al., 2010). However, the underlying signaling does not involve NMDARs as in PCs, rather, it depends on the activation of CP-AMPA and group 1 metabotropic glutamate receptors (Sambandan et al., 2010; Hainmüller et al., 2014).

A central finding of our present study is that GABA_B_R activation interferes with plasticity and markedly reduces LTP at this synapse. This observation converges with results from our prior work, demonstrating that GABA_B_R activation suppresses LTP at the recurrent excitatory input onto dendritic-targeting SOM-INs in the CA1 area (Booker et al., 2018). While these findings together may indicate an overarching principle of how GABA_B_Rs modulate synaptic plasticity in GABAergic INs, the underlying mechanisms are distinct: in CA1 SOM-INs, the suppression of LTP is dependent on GABA_B_R-mediated inhibition of postsynaptic L-type Ca^2+^ channels (Booker et al., 2018). In contrast, in DG PV-INs reduction of LTP involves the activation of the effector Kir3 channels. Thus, while the net effect of GABA_B_R activation may converge, modulation of plasticity is likely to be heterogenous depending on the IN type and region in terms of the underlying signaling mechanisms.

### 4.3 LTP induction and its modulation by GABA_B_R activation is similar in PV-BCs and PV-AACs

PV-INs comprise two major morphological types providing perisomatic inhibition: BCs preferentially targeting the soma and proximal dendrites of DGCs as well as other INs, and AACs selectively innervating the AIS of DGCs (Kosaka et al., 1987; Soriano and Frotscher, 1989; Buhl et al., 1994; Hosp et al., 2014; Elgueta and Bartos, 2019; Degro et al., 2024). While there is increasing evidence that BCs and AACs serve divergent functions in cortical networks (Viney et al., 2013; Dudok et al., 2021), in the DG most functional studies, including those focusing on synaptic plasticity, either did not systematically distinguish these two perisomatic inhibitory types or focused specifically on BCs (Bartos et al., 2001; Alle et al., 2002; Sambandan et al., 2010), therefore little is known about plasticity in AACs. Here we acquired and identified sufficiently large samples of these two types to assess whether differences exist in properties of their synaptic plasticity. In fact, recent findings indicate divergence in their active properties, such as discharge characteristics (Proddutur et al., 2023). However, we did not observe differences between BCs and AACs in the induction or magnitude of LTP at their MF synapse. Furthermore, the suppressing effect of GABA_B_R activation on plasticity at this synapse was found to be qualitatively and quantitatively similar between the two types.

### 4.4 Functional relevance and implications of GABA_B_ receptor modulation of plasticity in DG interneurons

GABA_B_Rs are widely expressed pre- as well as postsynaptically in both PCs and INs in the cortex (Kaupmann et al., 1998; Fritschy et al., 1999; Kulik et al., 2003, 2006, 2018). At the cellular level, they lower postsynaptic excitability and also reduce transmissions at both glutamatergic excitatory and GABAergic inhibitory synapses (Harrison et al., 1990; Otis et al., 1993). As a consequence, their activation is expected to dampen network activity (Booker et al., 2020). In the DG, however, GABA_B_R activation results in disinhibition and an enhanced network throughput, due to the overwhelming inhibitory effects on INs and GABA release (Mott and Lewis, 1991; Mott et al., 1993; Foster et al., 2013).

Beyond these immediate cellular and network effects, GABA_B_R activation has been shown to promote synaptic plasticity in DGCs (Mott and Lewis, 1991). The enhancement of plasticity in the DG network contrasts the direct cellular level inhibitory actions of GABA_B_Rs on PCs and their excitatory synapses (Sodickson and Bean, 1996; Lüscher et al., 1997; Chalifoux and Carter, 2010). At the circuit level, disinhibition of DGCs dominates, leading to progressive depolarisation and facilitation of NMDARs during the induction phase (Mott and Lewis, 1991). This mechanisms, however, is not specific to the DG circuit, but also observed in the CA1, and plausibly applies to other cortical networks (Davies et al., 1991). At a network level, disinhibition modulates the balance of excitation and inhibition by favouring excitation (Froemke et al., 2007; Donato et al., 2013). This is an important mechanism for learning, memory retrieval and circuit plasticity (Letzkus et al., 2015).

Our studies show that, in contrast to PCs, in INs GABA_B_R activation suppresses LTP at excitatory input synapses in the DG and the CA1 area (Booker et al., 2018; present results). This divergence in excitatory synaptic plasticity in these two classes of cortical neurons, PCs and INs, would result in a differential shift in their activation and recruitment to population activity. Such modulation of plasticity may occur during heightened oscillatory activity in the network which can, on the one hand, promote plastic changes and, on the other, result in spill-over of GABA from IN axon terminals, activating the extrasynaptically localized GABA_B_Rs (Scanziani, 2000; Kulik et al., 2003). This mechanisms has an implication for the long-term balance of excitation versus inhibition (E/I), tilting this balance towards excitation in the network.

Plasticity of the MF synapses onto PV-INs is thought to stabilize active DGC assemblies that encode spatial contextual information by silencing less active DGCs, and enhancing the signal to noise ratio in the system (Hainmüller et al., 2014). Limiting the recruitment of feedback inhibition might be nevertheless advantageous in the DG network to adjust the transfer of entorhinal inputs to the DGC output direct to the CA3, when larger assemblies are formed, to enhance the dynamic range along the main axis of the information flow.

In a pathophysiological context, the DG has been described as a protective “gate” against overt excitation reaching the hippocampus (Heinemann et al., 1992). Convergently, a recent optogenetic study showed that modulation inhibition of DGCs can halt the propagation of seizures in epileptic mice (Krook-Magnuson et al., 2015). While direct effects of GABA_B_R on DGCs are inhibitory, and can help dampen overall network activity, inhibition of GABAergic interneurons produce disinhibition and enhance rapid transfer through this network (Foster et al., 2013). Interestingly, GABA_B_R activation can also enhance the level of tonic inhibition via extrasynaptic GABA_A_Rs and reduce hyperexcitability of the network (Li et al., 2022). Our findings provide an additional piece of information to this complex picture and suggests that GABA_B_R modulation of plasticity onto PV-INs will act to reduce recruitment of rapid perisomatic inhibition. Thus, acute as well as long-term effects of these receptors convergently facilitate rapid informational flow, but enhance slow and tonic inhibition, plausibly with an overall stabilizing effect on the network without compromising information transfer.

## Data Availability

The raw data supporting the conclusions of this article will be made available by the authors upon request, without undue reservation.

## Footnotes

^1^ https://spider.science.strath.ac.uk/sipbs/software_ses.htm

^2^ https://github.com/neurodroid/stimfit

^3^ http://fiji.sc

^4^ https://www.neutracing.com
